# Drug development in psoriatic arthritis: a trialtrove-based landscape analysis (1999–2025)

**DOI:** 10.3389/fimmu.2026.1722103

**Published:** 2026-03-12

**Authors:** Gang Wang, Jun Li, Xinling Su, Zhuangwei Fang, Yuting Xu, Juan Zheng, Hao Liu, Ning Wang, Liping Huang

**Affiliations:** Department of Rehabilitation Medicine, the First Medical Center, Chinese People’s Liberation Army (PLA) General Hospital, Beijing, China

**Keywords:** clinical trial, drug, landscape, psoriatic arthritis, trialtrove database

## Abstract

**Background:**

Psoriatic arthritis (PsA) is a heterogeneous, immune-mediated disease affecting joints, entheses, the axial skeleton, and skin. Although many targeted therapies have emerged, unmet needs remain in musculoskeletal control, comorbidity management, and durable remission.

**Objective:**

To map the contemporary clinical-trial landscape of PsA pharmacotherapy and place these data in the context of current evidence, treatment guidelines, regulatory changes, and translational advances.

**Methods:**

We analyzed Citeline Trialtrove data on 587 interventional PsA drug trials initiated from 1999 to 2025. Structured variables captured annual trial starts by phase, operational status and outcomes, geographic distribution, funding sources, investigated drugs, molecular targets, and primary endpoints. We characterized trends using descriptive statistics and graphical summaries. To strengthen interpretation of temporal trends, we additionally prespecified count-based trend testing (Poisson/negative binomial regression) and conducted era-stratified summaries (1999–2010 vs 2011–2025) to reflect systemic changes in regulation and transparency.

**Results:**

Trial activity was higher after 2012, with increased early-phase programs and sustained phase III/IV development. Most studies reached completion (82.5%); 22.7% disclosed positive outcomes, while many were labeled undefined because of incomplete or non-standardized reporting. Terminations were driven mainly by business decisions rather than lack of efficacy. The United States (US) and China had the highest absolute trial participation, with Europe providing strong multicenter coordination; population-standardized participation (trials per 10 million population) was higher in the US than in China. Funding was diverse: academic institutions (35.3%) and top-20 pharmaceutical companies (32.9%) predominated, alongside smaller industry and generic sponsors. TNF inhibitors such as adalimumab and etanercept were the most frequently tested agents, but substantial activity involved newer mechanisms including IL-17/IL-23, JAK1/TYK2, and PDE4 pathways.

**Conclusions:**

Over the past two decades PsA drug development has broadened from TNF blockade to multiple targeted axes, supported by balanced academic–industry sponsorship and a rapidly expanding global footprint. Yet challenges remain—heterogeneous endpoints, incomplete outcome reporting, modest musculoskeletal efficacy in some novel classes, and the need to integrate cost considerations as biosimilars and generics enter routine care. Future trials should prioritize harmonized, domain-specific outcomes, precision patient selection, long-term safety (especially for JAK-pathway inhibitors), and pragmatic, treat-to-target designs to translate mechanistic advances into sustainable, patient-centered therapy.

## 1 Introduction

Psoriatic arthritis (PsA) is a chronic inflammatory disease characterized by diverse musculoskeletal manifestations, including peripheral arthritis, axial involvement, dactylitis, and enthesitis (1). Psoriasis affects approximately 2–3% of the global population, and between 6–42% of these patients develop inflammatory arthritis (2). Beyond joint damage, PsA impairs daily function and diminishes quality of life; research reveals that the impact of PsA extends to various aspects of life, including activities of daily living, physical, and emotional aspects, such as fatigue, sleep disturbance, anxiety and depression (3). Spontaneous remission of psoriatic arthritis is extremely rare (4), and, if untreated, it can cause irreversible structural damage (5). As with other chronic inflammatory disorders, PsA and its associated comorbidities (such as cardiovascular disease) contribute to long-term disability and reduced lifespan (6).

Despite advances in therapy, PsA remains a heterogeneous and often difficult-to-treat condition. Diagnostic delays, variable treatment responses, and comorbidities hinder consistent achievement of sustained remission (7). International societies including European Alliance of Associations for Rheumatology (EULAR), the Group for Research and Assessment of Psoriasis and Psoriatic Arthritis (GRAPPA), and the American College of Rheumatology/National Psoriasis Foundation (ACR/NPF) advocate a treat-to-target (T2T) strategy emphasizing remission or low disease activity, while also recommending that therapeutic choices be tailored to disease domains and patient safety profiles. The most recent EULAR 2024 update underscores increasing attention to drug safety in clinical decision-making and shared decision-making frameworks (8). Meanwhile, building on prior recommendations, the GRAPPA 2021 update adopted a Grading of Recommendations Assessment, Development and Evaluation (GRADE)-informed, domain-oriented framework and explicitly added principles on biosimilars and tapering. It also underscored patient-prioritized outcomes (pain, fatigue) and the need to define outcome sets for axial PsA and improve enthesitis assessment. In parallel, EULAR recommendations converge on domain-tailored therapy but differ in aspects of sequencing, reflecting both new agents and evolving safety considerations (9). Over the past two decades, PsA has become the rheumatologic disease with the broadest therapeutic armamentarium (10). Mechanistic diversity has expanded from tumor necrosis factor (TNF) blockade to inhibition of IL-17, IL-23, JAK/TYK2, and PDE4 pathways. Regulatory changes, including JAK inhibitor boxed warnings and evolving biosimilar policies, have further shaped both clinical practice and drug accessibility. Although TNF-α neutralizing agents remain the current first-line therapy for PsA, they are associated with increased risks of opportunistic infections, reactivation of latent tuberculosis, and malignancies, which can hinder long-term use (11). Nonetheless, a substantial subset of patients remain symptomatic and functionally impaired despite multiple biologic or targeted synthetic disease-modifying antirheumatic drugs (DMARDs), underscoring the persistence of unmet needs (12).

Here we present a Trialtrove-based descriptive analysis of interventional PsA drug trials conducted between 1999 and 2025. By integrating trial activity with outcomes, geography, sponsorship, agents/targets, and primary endpoints, and by contextualizing with external literature, we map the therapeutic landscape and highlight opportunities to refine strategies for next-generation PsA therapies.

Research gap and practical problem. Although PsA therapeutics have diversified rapidly, there remains limited integrative mapping that simultaneously evaluates long-horizon trial activity, endpoint heterogeneity, reporting completeness (e.g., “Undefined” outcomes), geographic and sponsor ecosystems, and mechanism diversity, factors that directly affect cross-trial comparability and the translation of T2T principles into pragmatic trial design.

## 2 Materials and methods

### 2.1 Data source and scope

We used curated extracts from Citeline Trialtrove, a commercial analyst-curated clinical trial intelligence platform aggregating information from over 60,000 sources with both real-time updates and historical coverage. Trialtrove integrates multiple public and proprietary sources (including, but not limited to, trial registries, publications, conference abstracts, press releases, and sponsor disclosures), enabling broad coverage beyond any single registry. The data freeze and extraction were both completed on September 5, 2025. The seven structured tables summarized in this review cover: (1) annual trial counts by phase, (2) overall trial status and outcomes, (3) country participation (single-center vs multicenter), (4) funder type, (5) drugs by trial count, (6) molecular targets by trial count, and (7) primary endpoints.

### 2.2 Data integrity, de-duplication, and quality control

Because registry entries and secondary sources can contain errors or discordant fields, and because completion status may differ across registries for the same trial, we implemented the following internal quality control (QC) steps:

Record linkage and de-duplication: When multiple records mapped to the same underlying study (e.g., multiple registries, sub-studies, site expansions), we prioritized the most complete master record and consolidated linked entries to avoid double counting, using registry identifiers (when available) and concordance of title/agent/phase/timeline/country fields.

Consistency checks: We screened for implausible or internally inconsistent combinations (e.g., phase incompatible with endpoint type; completed status without a plausible start year) and resolved discrepancies by defaulting to the master record.

Plausibility review: We reviewed extreme values (e.g., unusually high endpoint frequencies) against underlying structured fields to confirm they reflected early-phase pharmacokinetic clustering rather than duplication artifacts.

We did not perform a full independent re-extraction from each registry (ClinicalTrials.gov; the EU Clinical Trials Register [EUCTR]/EudraCT; and the Chinese Clinical Trial Registry [ChiCTR]) in this landscape review; instead, we explicitly acknowledge cross-registry discordance as a limitation and interpret registry-dependent fields (notably outcomes) cautiously (13).

### 2.3 Eligibility and definitions

We included interventional drug trials enrolling patients with PsA across phases I–IV and all sponsors. Pediatric-only, non-pharmacologic (e.g., surgery, rehabilitation), and non-interventional studies were excluded. Where multiple records mapped to the same trial (e.g., multiple registries, sub-studies, site expansions), we applied a de-duplication rule privileging the most complete master record and consolidated linked entries to avoid double counting.

### 2.4 Status and outcomes

Operational status labels (Completed, Open, Planned, Terminated, Closed) and outcome labels for completed studies (Positive, Negative, Indeterminate, Unknown, Undefined) were taken as-is from Trialtrove without re-adjudication against primary publications. “Undefined” indicates absence of a normalized outcome label in Trialtrove (e.g., incomplete reporting or non-standard aggregation). Trialtrove’s structured outcome fields do not provide sufficient metadata to reliably decompose the ‘Undefined’ category into specific underlying causes (e.g., absence of public results reporting versus non-standardized aggregation across sources). Therefore, we did not quantify these components and treated ‘Undefined’ as missing/insufficiently standardized outcome information. Termination reasons reflect Trialtrove categories.

To improve interpretability, we provide the following operational interpretations of Trialtrove’s normalized labels: Positive indicates Trialtrove labeled the outcome as positive based on available public information; Negative indicates a negative label; Indeterminate indicates mixed/unclear results; Unknown indicates insufficient information to classify; and Undefined indicates the lack of a normalized outcome label in the structured field. We did not re-adjudicate against primary publications to avoid subjective reclassification bias and because the scope of this study is a landscape mapping rather than an effectiveness meta-analysis.

### 2.5 Variables and analysis

**Figure 1:**
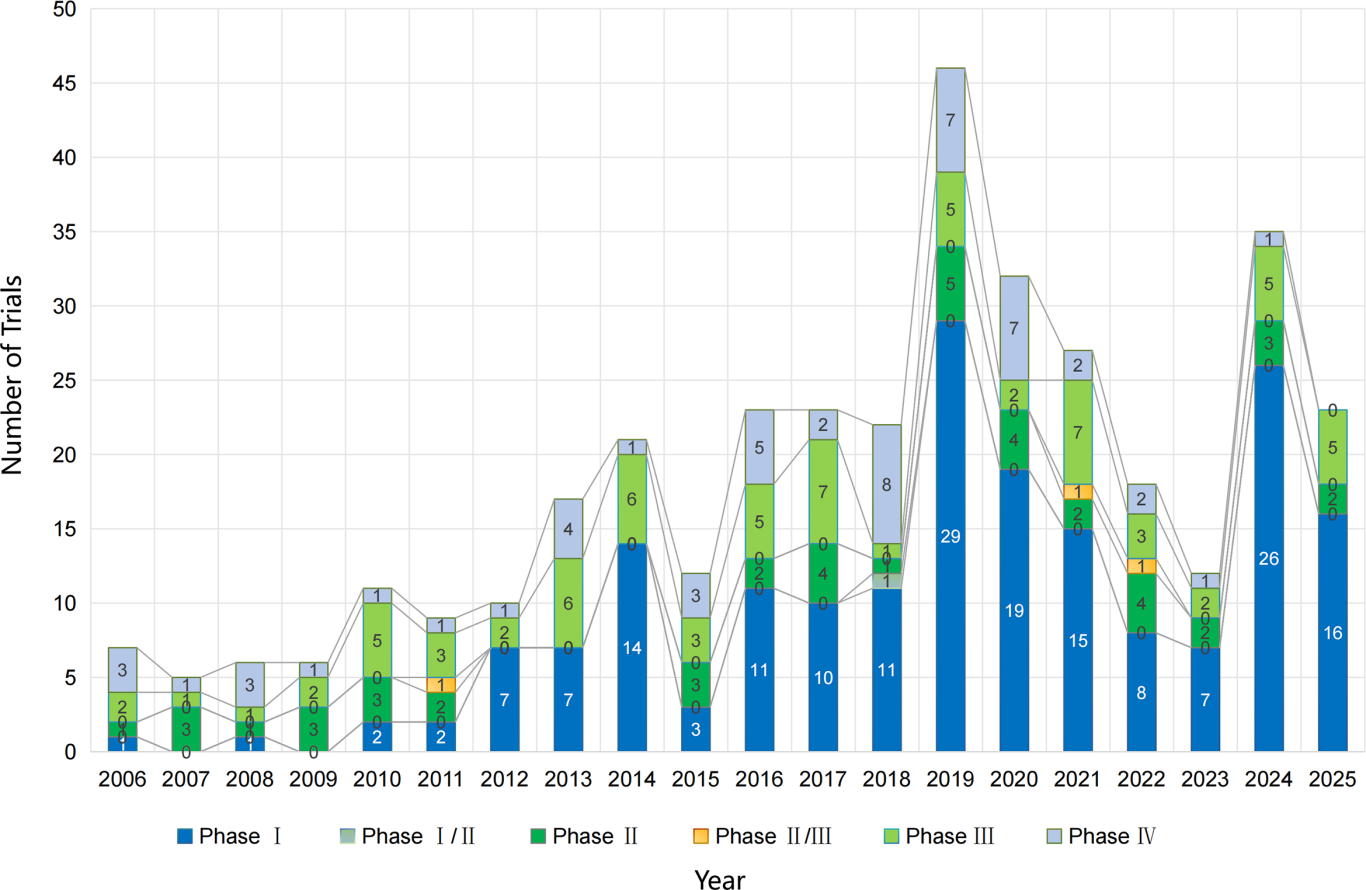
Annual number and phase distribution of PsA drug trial initiations (1999–2025).

**Figure 2:**
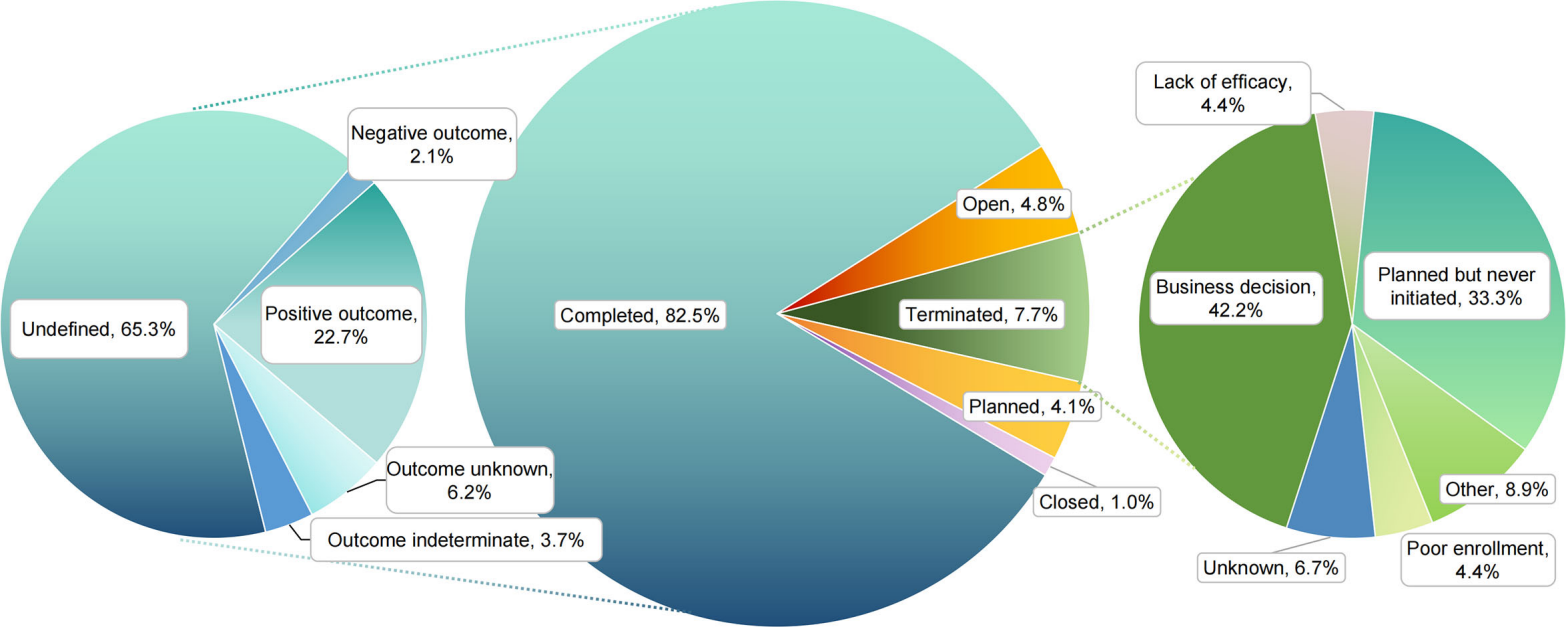
Operational status and disclosed outcomes of PsA clinical trials.

**Figure 3:**
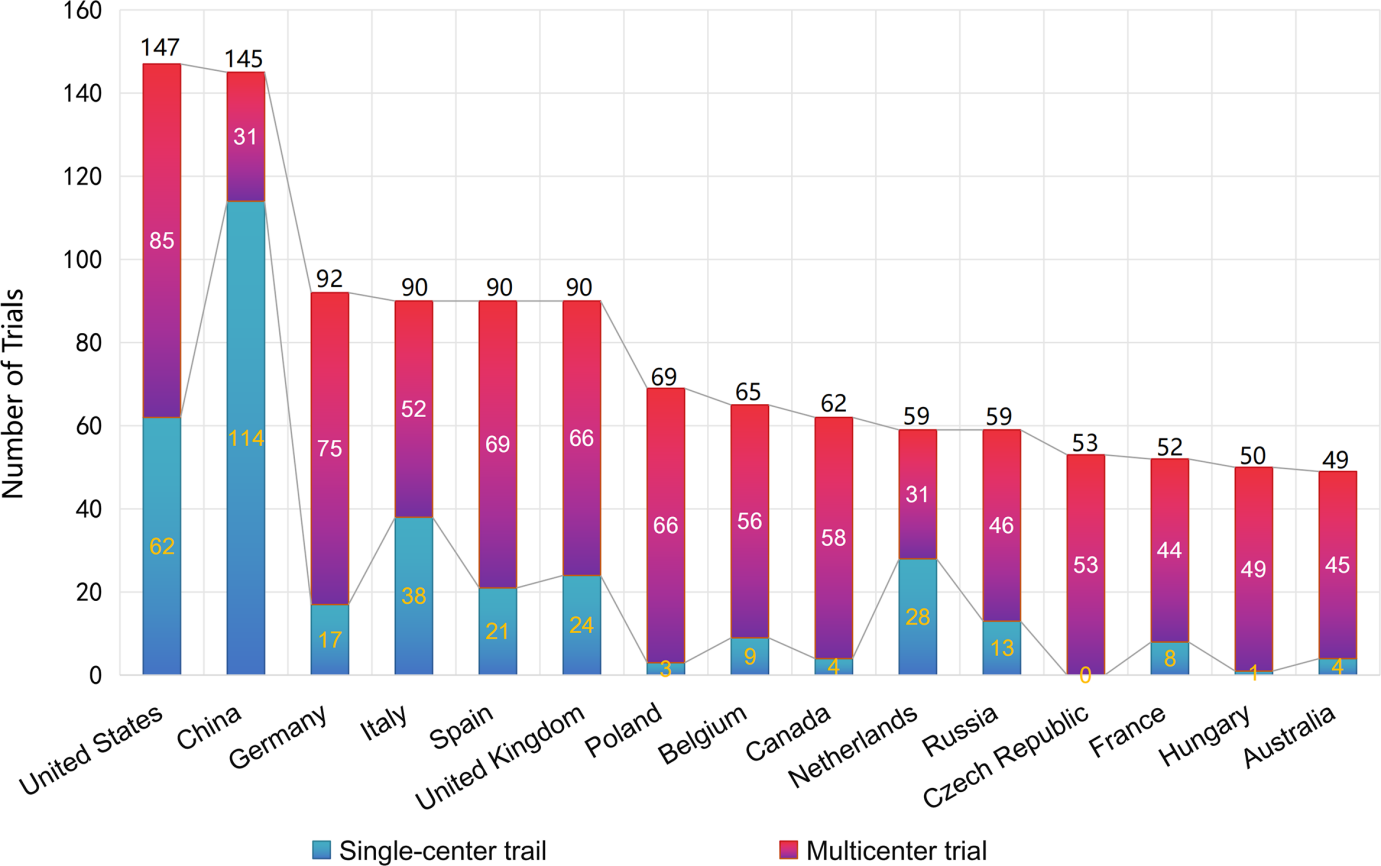
Country participation and trial center type (single vs multicenter) in PsA clinical trials.

**Figure 4:**
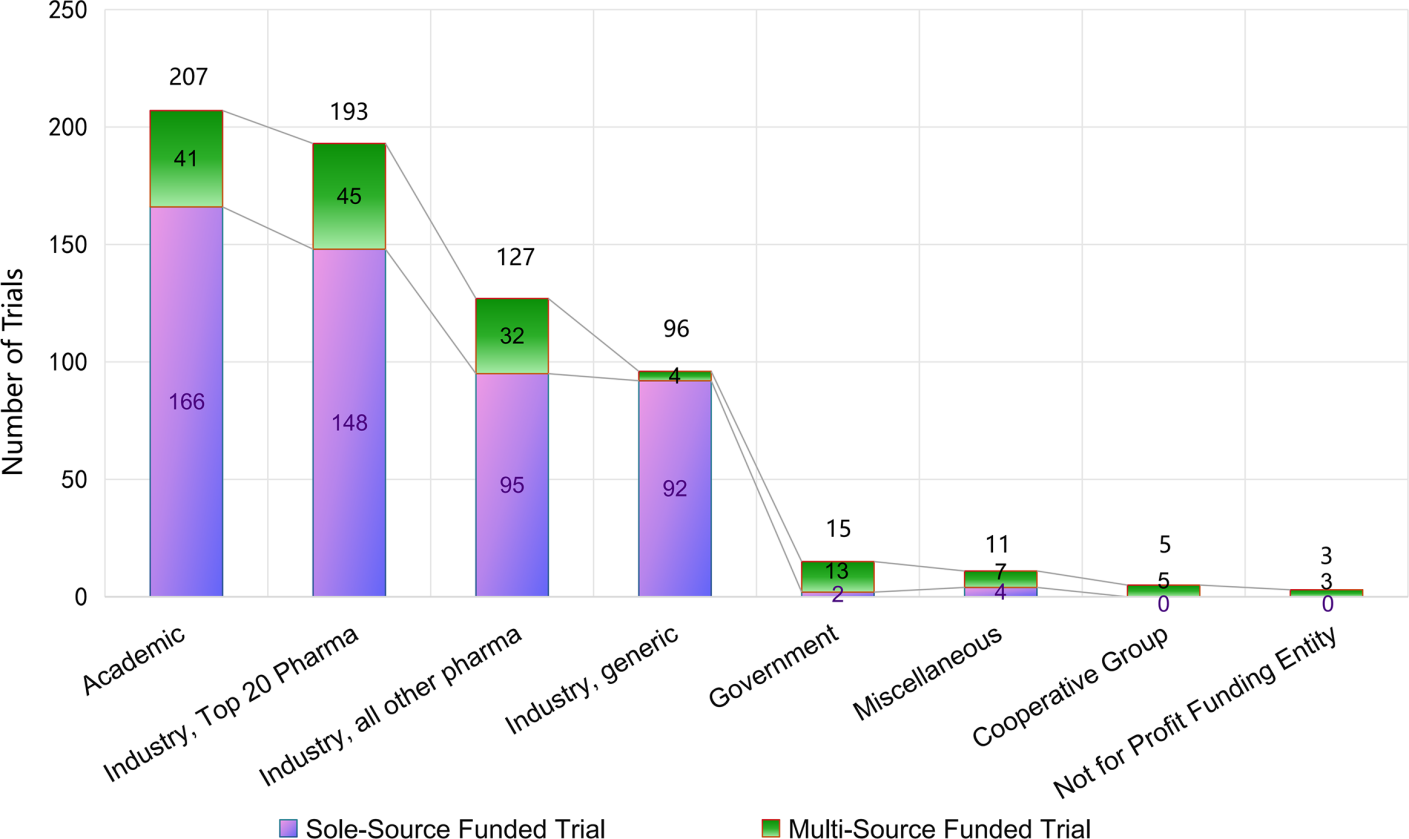
Distribution of funding sources for PsA clinical trials.

**Figure 5:**
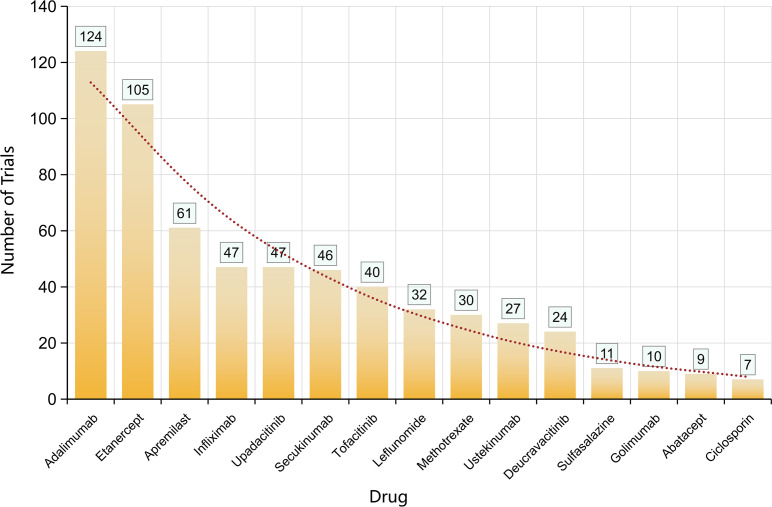
Most frequently investigated drugs in PsA clinical trials.

**Figure 6:**
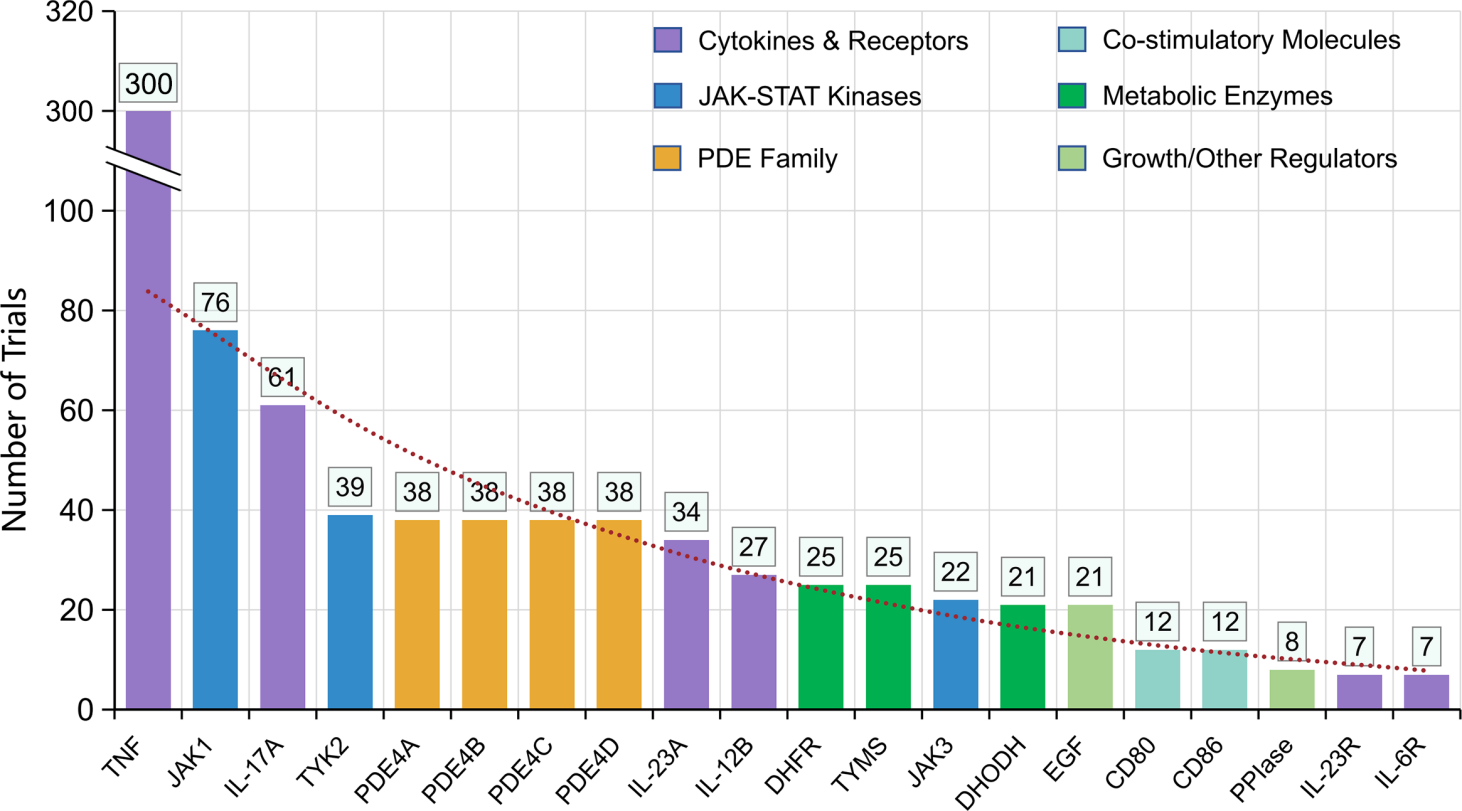
Distribution of molecular targets in PsA clinical trials. TNF, tumor necrosis factor; JAK1, Janus kinase 1; IL-17A, interleukin 17A; TYK2, tyrosine kinase 2; PDE4, phosphodiesterase-4; IL-23A, interleukin 23A; IL-12B, interleukin 12B; DHFR, dihydrofolate reductase; TYMS, thymidylate synthetase; JAK3, Janus kinase 3; DHODH, dihydroorotate dehydrogenase; EGF, epidermal growth factor receptor; CD80, CD80 molecule; CD86, CD86 molecule; PPIase, peptidylprolyl isomerase A; IL-23R, interleukin 23 receptor; IL-6R, interleukin 6 receptor.

**Figure 7:**
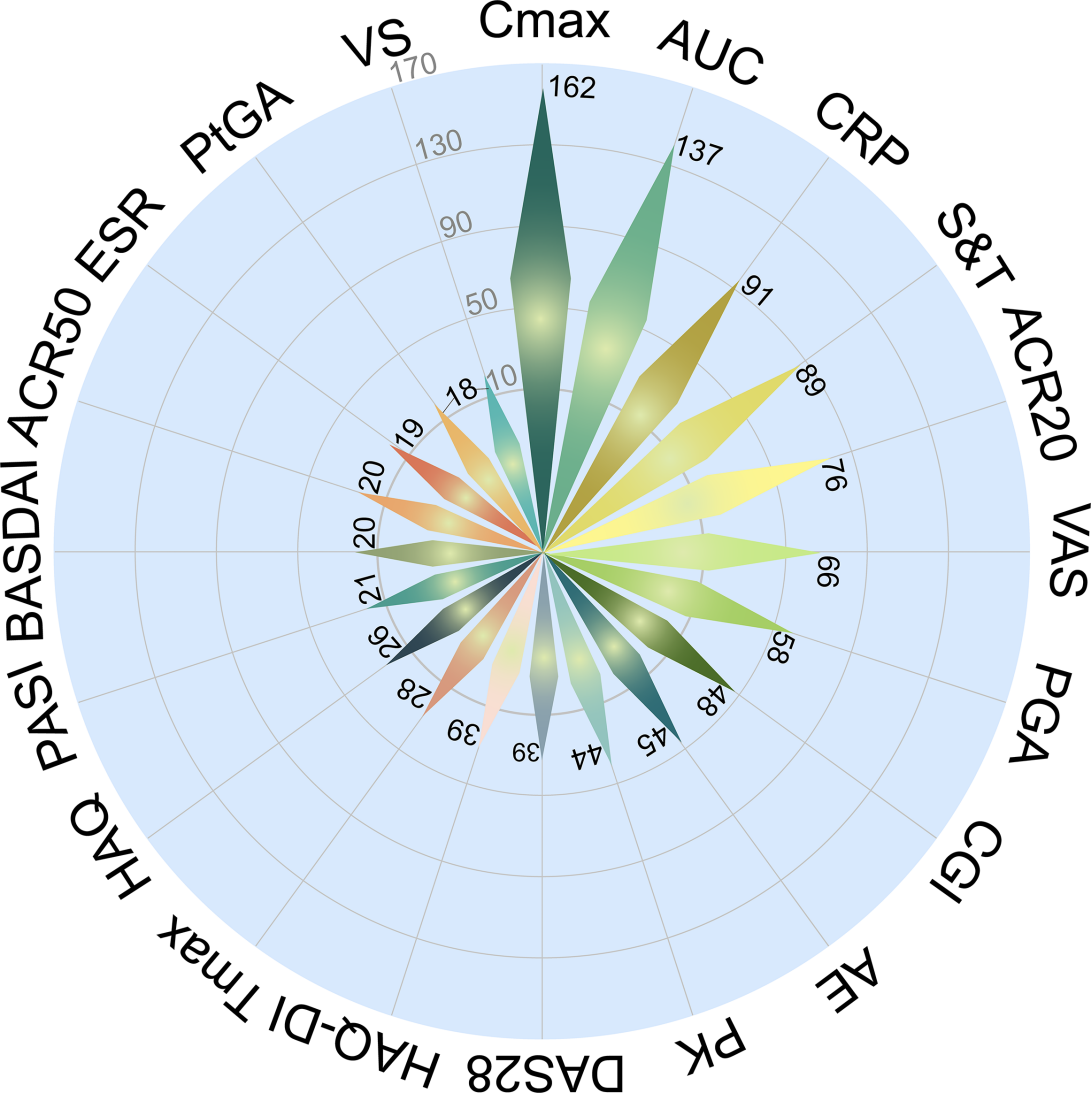
Primary endpoints utilized in PsA clinical trials. Cmax, maximum plasma concentration; AUC, area under the concentration-time curve; CRP, C-reactive protein; S&T, signs and tenderness; ACR20/50, American College of Rheumatology 20%/50% response; VAS, visual analogue scale; PGA, Physician Global Assessment; CGI, Clinical Global Impression; AE, adverse event; PK, pharmacokinetics; DAS28, Disease Activity Score in 28 joints; HAQ-DI, Health Assessment Questionnaire Disability Index; Tmax, time to reach maximum plasma concentration; HAQ, Health Assessment Questionnaire; PASI, Psoriasis Area and Severity Index; BASDAI, Bath Ankylosing Spondylitis Disease Activity Index; ESR, erythrocyte sedimentation rate; PtGA, Patient Global Assessment; VS, vital signs.

Temporal activity was defined by study start year per Trialtrove. Country participation distinguished single-center vs multicenter roles. Sponsorship categories (academic, top-20 pharmaceutical, other pharmaceutical, generic industry, government, cooperative groups, not-for-profit, miscellaneous) were non-mutually exclusive; co-funded trials contributed to multiple categories, so category totals exceed the number of unique trials. Primary endpoints were summarized as recorded in Trialtrove; field nomenclature is reported verbatim. Analyses were descriptive with no imputation. **Figures 1**–**7** display the seven table domains; where appropriate, multi-panel layouts are used.

### 2.6 Missing data handling and sensitivity analyses

We did not impute missing values. For outcome reporting, “Undefined” and “Unknown” were treated as missing/insufficiently standardized outcome information rather than efficacy-neutral outcomes. We summarized the frequency of missing/undefined outcome labels and, where relevant, interpreted completed-trial outcome distributions both (i) in the full set of completed trials and (ii) in sensitivity analyses restricted to trials with explicitly labeled outcomes (Positive/Negative/Indeterminate). We contextualize reporting incompleteness in light of ongoing global initiatives to improve registry summary-results reporting (14).

### 2.7 Statistical analysis (trend testing and era stratification)

To support interpretation of time trends in annual trial starts (overall and by phase), we modeled annual counts using negative binomial regression with a log link to account for overdispersion. We report incidence rate ratios (IRRs) with 95% confidence intervals.

Given the long study window and systemic changes in transparency/regulation (e.g., implementation of the EU Clinical Trials Regulation/CTIS on 31 Jan 2022 (15)), we additionally conducted era-stratified analyses comparing 1999–2010 versus 2011–2025.

### 2.8 Bias and limitations (methods-level)

Because Trialtrove integrates multiple public and proprietary sources, coverage and metadata quality may vary by region and sponsor. Publication lag and registry under-reporting can elevate “Undefined” outcome proportions. Our analysis did not perform risk-of-bias assessment or infer comparative effectiveness.

## 3 Results

In total, we identified 587 eligible interventional PsA drug trials initiated between 1999 and 2025; unless otherwise specified, the results below summarize these studies using descriptive statistics across the seven structured domains (phase-by-year, status/outcomes, geography, sponsorship, agents, targets, and primary endpoints) and correspond to **Figures 1**–**7**.

### 3.1 Time trends by phase (1999–2025)

The PsA pipeline shows intermittent activity through 2010, followed by a step-change upward after 2012, coinciding with the maturation of non-TNF mechanisms. Phase III activity became sustained from 2013 onward; Phase I starts surged in 2014, 2016–2019, and 2024–2025, indicating renewed early-stage diversification. Phase IV activity persisted, consistent with post-marketing commitments and label expansions.

Key numbers: Peaks included 2019 (Phase I: 29; Phase IV: 7) and 2024 (Phase I: 26; Phase III: 5; Phase IV: 1). In 2025, programs remained robust (Phase I: 16; Phase III: 5) (**Figure 1**).

In the subset with recorded start year and phase (n = 395), the annual trial-start rate in 2011–2025 was 4-fold higher than in 1999–2010 (IRR 4.06, 95% CI: 3.11–5.29; p < 0.001). This increase was largely driven by early-phase expansion (Phase I: IRR 24.67, 95% CI: 10.9–55.7; p < 0.001), while Phase III and Phase IV initiations also increased significantly (Phase III: IRR 2.36, 95% CI: 1.49–3.72; Phase IV: IRR 2.12, 95% CI: 1.30–3.47). The phase composition differed markedly between eras (early vs mid vs late; chi-squared test p < 0.001), with a substantially higher share of early-phase starts after 2011.

Additional note on denominators. The overall cohort included 587 eligible interventional PsA drug trials (1999–2025). However, the phase-by-year initiation analysis (**Figure 1**) required both a recorded study start year and a defined trial phase in Trialtrove; therefore, **Figure 1** summarizes the subset with complete start year and phase information (n = 395). Trials with an unknown/unreported phase and/or missing start year (n = 192) were not shown in **Figure 1** but were retained in other analyses using variable-specific available-case denominators.

### 3.2 Operational status and disclosed outcomes

Across 587 trials: completed constituted 82.5% (n = 484); open 4.8% (n = 28); planned 4.1% (n = 24); terminated 7.7% (n = 45); closed 1.0% (n = 6). Among completed studies (n = 484), outcomes were mostly undefined (65.3%; n = 316), with positive outcome in 22.7% (n = 110), indeterminate 3.7% (n = 18), negative 2.1% (n = 10), unknown 6.2% (n = 30).

Terminations. Most were business decisions (42.2%; n = 19), followed by planned but never initiated (33.3%; n = 15). Lack of efficacy and poor enrollment each accounted for 4.4% (n = 2), with unknown 6.7% (n = 3) and other 8.9% (n = 4) (**Figure 2**).

### 3.3 Geography of development

The US (n = 147) and China (n = 145) led global participation, but roles differed: China’s contribution was predominantly from single-center trials (114 single-center vs 31 multicenter trials), whereas the US skewed more multicenter (85 vs 62 single-center). European countries (Germany 92; Italy 90; Spain 90; UK 90) exhibited multicenter predominance, emphasizing Europe’s coordinating role for cross-national studies (**Figure 3**).

To complement absolute counts, we computed a population-standardized indicator (trials per 10 million population). Using World Bank population totals (US: 340,110,988 in 2024; China: 1,408,975,000 in 2024), trial participation corresponds to approximately 4.3 trials per 10 million for the US and 1.0 trials per 10 million for China over the study period. We interpret these per-capita comparisons cautiously because cross-country registry practices and transparency requirements can influence apparent participation and data completeness (15).

### 3.4 Sponsor ecosystem

The funding landscape remained diverse. Academic institutions were the largest contributors, supporting 207 trials (35.3%), followed by top-20 pharmaceutical companies with 193 trials (32.9%). Other pharmaceutical sponsors (non-top-20) accounted for 127 trials (21.6%), while the generic drug industry funded 96 trials (16.4%). Government agencies, miscellaneous entities, cooperative groups, and not-for-profit organizations together contributed a smaller share (ranging from 3 to 15 trials each). Because many studies were co-sponsored, the sum of trials by funding source exceeded the total trial count of 587 (**Figure 4**).

### 3.5 Agents most frequently studied

The most investigated drugs were: adalimumab (n = 124), etanercept (105), apremilast (61), infliximab (47), upadacitinib (47), secukinumab (46), tofacitinib (40), leflunomide (32), methotrexate (30), ustekinumab (27), deucravacitinib (24), followed by sulfasalazine, golimumab, abatacept, ciclosporin. This hierarchy reflects legacy TNF programs plus expansion into PDE4 (apremilast), IL-12/23 and IL-23 (ustekinumab, p19 agents), JAK1 (upadacitinib), and TYK2 (deucravacitinib) (**Figure 5**).

### 3.6 Molecular targets

TNF dominated the target landscape (n = 300). Second-tier targets included JAK1 (76), IL-17A (61), TYK2 (39), and the PDE4 isoforms (38 each for PDE4A/B/C/D), followed by IL-23A (p19), IL-12B (p40), DHFR/TYMS (classical conventional synthetic DMARDs mechanisms), JAK3, DHODH, EGF, CD80/CD86, PPIase, IL-23R, and IL-6R. Overall, the field shows broadening beyond TNF toward cytokine, intracellular kinase, and metabolic pathways (**Figure 6**).

### 3.7 Primary endpoints

Analysis of 587 interventional PsA trials revealed a diverse set of primary endpoints, reflecting both pharmacokinetic measures and clinical outcomes. Pharmacokinetic readouts were highly represented, with maximum plasma concentration (Cmax, n = 162) and area under the concentration-time curve (AUC, n = 137) ranking as the two most frequently specified primary endpoints, followed by time to reach maximum plasma concentration (Tmax, n = 28). Inflammatory biomarkers also appeared prominently, including C-reactive protein (CRP, n = 91) and erythrocyte sedimentation rate (ESR, n = 19).

Standard clinical efficacy endpoints were widely employed. Composite indices such as American College of Rheumatology 20% (ACR20, n = 76), American College of Rheumatology 50% (ACR50, n = 20), and Disease Activity Score in 28 joints (DAS28, n = 39) were frequent, alongside functional measures like Health Assessment Questionnaire Disability Index (HAQ-DI, n = 39) and Health Assessment Questionnaire (HAQ, n = 26). Pain and patient-reported outcomes featured strongly, including visual analogue scale (VAS, n = 66), Physician Global Assessment (PGA, n = 58), Patient Global Assessment (PtGA, n = 18), and global scales such as Clinical Global Impression (CGI, n = 48). Dermatologic indices were less frequent but notable, with Psoriasis Area and Severity Index (PASI, n = 21) capturing skin responses, while Bath Ankylosing Spondylitis Disease Activity Index (BASDAI, n = 20) reflected axial disease burden.

Safety and tolerability endpoints were consistently represented: adverse events (AE, n = 45) and pharmacokinetics (PK, n = 44) appeared across multiple development phases. Other clinical markers included signs and tenderness (S&T, n = 89) and vital signs (VS, n = 18), typically linked to functional or physician-based assessments (**Figure 7**).

## 4 Discussion

### 4.1 Temporal trends in PsA drug development

Our longitudinal analysis of interventional PsA trials (1999–2025) demonstrates marked evolution in the treatment pipeline. Early years (1999–2010) exhibited sparse trial initiation across all phases, in keeping with legacy reliance on TNF inhibitors and conventional DMARDs. However, beginning in 2012, a discernible uptick in trial activity—particularly Phase III and Phase I—coincided with the maturation of non-TNF target pathways. Phase I trials surged notably in 2014, 2016–2019, and again in 2024–2025, indicating renewed early-stage drug exploration. Sustained Phase III activity after 2013 reflects increasing regulatory readiness for novel mechanisms.

These trends align with recent reviews highlighting diversification beyond TNF inhibition into IL-17/IL-23, JAK/TYK2, and PDE4 pathways (16). The proliferation of small-molecule oral agents and biologics targeting diverse cytokines underscores a paradigm shift in PsA drug development.

Implications. The resurgence in early-phase studies suggests sustained industry and academic interest in addressing unmet needs, including inadequate responses to existing therapies, domain-specific manifestations (e.g., enthesitis, axial disease), and safety concerns. Future trials may benefit from adaptive designs and biomarker-driven stratification to further accelerate early development.

### 4.2 Trial outcomes and termination patterns

Among 587 interventional trials, 82.5% reached completion, but only 22.7% yielded a positive outcome; most outcomes were classified as “Undefined” (65.3%). The predominance of “Undefined” likely reflects incomplete publication or non-standardized aggregation rather than inherent inefficacy. Terminations (7.7%) were primarily driven by business decisions (42.2%) or failure to initiate (33.3%), with actual efficacy/failure reasons such as lack of efficacy or poor enrollment accounting for < 10%. Collectively, these patterns suggest portfolio reprioritization and operational factors outweighed objective futility in trial cessation. This distribution likely reflects publication bias and incomplete registry reporting. It also suggests that strategic portfolio decisions were a bigger driver of termination than scientific futility. Prior literature cautions that meta-analyses based solely on published results inflate perceived success rates in PsA and other inflammatory diseases. The high proportion of “Undefined” underscores the need for more transparent reporting and post-trial data sharing.

Implications. Academic and regulatory bodies should advocate for full outcome disclosure, even for unsuccessful or halted trials, to improve evidence synthesis and avoid wasted efforts. Integrating trial registries with publication incentives and real-time results updates could enhance transparency and scientific integrity. This concern aligns with updated WHO guidance (2025) describing minimum items needed for interpretable summary results reporting in registries, emphasizing timely and complete disclosure to reduce research waste and selective reporting (14).

### 4.3 Geographic distribution and global collaboration

The US and China continue to represent the two most active regions in PsA clinical development. The U.S. has historically favored multicenter designs, whereas most Chinese studies have been conducted at single institutions. In Europe, countries such as Germany, Italy, Spain, and the UK have consistently organized multicenter programs, highlighting the region’s strong infrastructure for international collaboration.

These differences broadly reflect global research dynamics: the U.S. remains a leader in trial coordination and multinational partnerships, while China has rapidly expanded its domestic trial base through single-site operations. Europe’s network-oriented model facilitates standardized protocols and region-wide patient recruitment, which is especially valuable for ensuring diverse representation.

Differences in PsA prevalence help explain where clinical trial activity is concentrated. A recent systematic review reported a global prevalence of about 112 cases per 100,000 adults, with higher pooled estimates in Europe (175/100,000) and North America, while prevalence appeared lower in Asia and South America, though data from many regions remain sparse and methodologically heterogeneous (17).

In China, insights from the Chinese Registry of Psoriatic Arthritis (CREPAR) provide the first large-scale, prospective description of affected patients. Among 1,074 patients enrolled between 2018 and 2021, peripheral arthritis was the most common presentation (86.5%), axial disease was present in 39.9%, and more than half of patients exhibited multiple musculoskeletal domains at onset (18). Disease activity was generally higher than that reported in European registries, and the use of biologic DMARDs was comparatively lower (29.1%). These findings suggest that, despite relatively limited epidemiologic prevalence data, the absolute number of patients and the expanding registry infrastructure are creating momentum for rapid growth of PsA trials in China.

Multicenter designs, particularly those involving sites across different healthcare systems in the U.S. and Europe (and with an expanding number of global sites), enhance external validity by recruiting more heterogeneous patient populations (19). However, existing evidence also shows that racial and ethnic representation remains highly unbalanced in PsA RCTs, suggesting that geographic spread alone does not fully ensure generalizability (20).

For PsA specifically, where disease heterogeneity spans joint, skin, enthesitis, and axial domains, the implications are acute. Multicenter, multi-regional participation allows not only faster recruitment but also more representative sampling for subgroup and domain-specific analyses. This aligns with the GRAPPA 2021 framework advocating domain-driven outcome assessment and strengthens the ability to transport trial findings across different ethnic and healthcare contexts (21). Future PsA trial designs should therefore integrate multicenter structures with prespecified plans for domain- and region-stratified analyses, while balancing efficiency, cost, and methodological rigor.

Implications. Expanding international, multicenter partnerships—including within Asia and South America—remains crucial to improve generalizability and accelerate recruitment. Building capacity for larger multicenter platforms in emerging research regions could reduce inequities in trial participation. Importantly, epidemiologic burden, affordability, and evolving biosimilar policies will continue to dictate both the pace and geographic spread of PsA clinical development.

Per-capita standardization suggests higher trial participation density in the US than China, but cross-country comparisons remain sensitive to registry practices and transparency regimes.

### 4.4 Sponsor and funding ecosystem

Our updated analysis of funding sources highlights a genuinely pluralistic sponsorship ecosystem. Academic institutions contributed over one-third of all PsA trials (35.3%), underscoring their central role in initiating mechanistic studies and investigator-led projects. Top-20 pharmaceutical companies closely followed, accounting for nearly one-third of trials (32.9%), primarily driving pivotal late-phase programs and global commercialization efforts. Mid-sized and smaller pharmaceutical companies together supported 21.6% of trials, reflecting both innovation in niche mechanisms and regional development activities. The generic drug sector, responsible for 16.4%, suggests a strong focus on biosimilar evaluation and cost-sensitive strategies.

Although government agencies, not-for-profit funders, and cooperative groups represented only a small fraction of total sponsorship, their involvement is noteworthy. These entities often support independent or comparative effectiveness research, which can complement industry-led initiatives. The presence of co-funding across categories indicates that multi-stakeholder collaboration is already common practice.

Implications. The balance between academic and industry support demonstrates mutual alignment: academia ensures scientific breadth and early translational exploration, while industry provides the resources and infrastructure necessary for large-scale validation. Expanding the role of government and not-for-profit sponsors could help address areas that are less commercially attractive—such as comparative head-to-head studies, trials in resource-limited settings, or research into long-term safety. Clearer disclosure of funding sources and stronger frameworks for public–private collaboration would also enhance transparency and trust in PsA research.

### 4.5 Drug-specific trends: legacy vs novel agents

The most frequently studied agents remain the legacy TNF inhibitors (adalimumab, etanercept, infliximab) and methotrexate, reflecting continued evaluation of established therapies in new contexts (e.g., different patient subsets, combination regimens). However, significant trial volume is evident for novel agents: apremilast (PDE4 inhibitor), upadacitinib (JAK1), secukinumab (IL-17A), ustekinumab (IL-12/-23), deucravacitinib (TYK2), and others.

This trajectory aligns with therapeutic evolution, where newer oral and biologic agents address previously unmet domains—such as skin or axial involvement—with differentiated safety or administration profiles.

A real-world study further suggested that, for patients failing an initial TNF inhibitor, switching to an IL-17 inhibitor may be more effective than cycling to a second TNF inhibitor (22). This observation highlights the importance of tailoring sequencing decisions based not only on mechanism of action but also on real-world treatment durability.

Costs are also an important aspect in patient management, and it is generally recommended to prescribe the less expensive drug when two agents show comparable efficacy and safety. Even when one mode of action demonstrates slightly better results in certain domains, a lower-cost alternative may remain preferable if efficacy loss is minimal. The upcoming availability of generic tofacitinib and apremilast is expected to reduce treatment costs and broaden access, especially in resource-limited settings. As highlighted by the 2024 EULAR update, therapeutic choice should balance efficacy, safety, and cost to optimize treat-to-target strategies (23).

Implications. Further head-to-head, real-world, and long-term safety studies comparing legacy and novel agents would help define optimal sequencing. Biomarker-driven stratification may also permit precision-targeted selection. Notably, the efficacy of upadacitinib has been shown to surpass that of the classic TNF-α inhibitor adalimumab in randomized trials. Nevertheless, clinicians must carefully weigh this superior efficacy against the increased risk of adverse events such as serious infections or hepatic enzyme elevations, particularly at higher doses (24).

### 4.6 Target evolution and mechanistic diversification

Target analysis reveals TNF remains dominant (300 trials), but newer targets: JAK1, IL-17A, TYK2, PDE4 isoforms, IL-23A, IL-12B-are well represented. Although IL-17 and IL-23 inhibitors deliver highly impressive skin clearance in PsA, improvements in musculoskeletal domains (peripheral arthritis, enthesitis, axial involvement) are often more modest in terms of speed, magnitude, or consistency. Several studies suggest that the pathways controlling skin inflammation may diverge from those driving joint, enthesis or axial disease, resulting in less robust joint outcomes despite excellent psoriasis responses (25). Many patients fail to respond adequately or sustain response to their first biologic DMARDs or targeted synthetic DMARDs, with even lower success rates upon subsequent lines of therapy (26). PsA is also more complex than rheumatoid arthritis, distinguished by its heterogeneous phenotype and involvement of multiple tissues, complicating uniform treatment strategies. Additionally, classical csDMARD targets (DHFR, TYMS), co-stimulatory molecules (CD80/CD86), EGF, and intracellular enzymes (PPIase, DHODH) illustrate exploration of alternate mechanistic schemas. Beyond JAK1 and TYK2 inhibitors, tofacitinib has demonstrated robust efficacy across multiple PsA domains—including peripheral arthritis, axial involvement, enthesitis, and dactylitis—making it a versatile option in difficult-to-treat cases (27). These data reinforce the role of JAK inhibitors as an important class for patients with multi-domain disease who are refractory to TNF inhibitors.

Recent reviews highlight the role of JAK-STAT signaling, underscore TYK2 inhibitor promise, and note trials of IL-17/IL-23 bispecific agents (28–30). The presence of PDE4 trials aligns with drugs like apremilast showing modest efficacy but favorable safety (31).

Implications. Future research should continue exploring combination targeting strategies and domain-specific mechanistic matching. Translating molecular understanding into clinical decision algorithms (e.g., JAK inhibitors for predominant enthesitis) could enhance treatment personalization.

### 4.7 Endpoint heterogeneity and harmonization needs

The spectrum of primary endpoints is broad: pharmacokinetic measures (Cmax, AUC, Tmax), biomarkers (CRP, ESR), composite rheumatologic (ACR20/50, DAS28), functional indices (HAQ-DI, HAQ), patient-reported outcomes (VAS, PGA, PtGA, CGI), dermatologic (PASI), and axial/fatigue scales (BASDAI, S&T, VS).

This heterogeneity reflects phase-specific endpoints (e.g., pharmacokinetics in early trials, clinical efficacy later) and domain coverage, but it hampers cross-trial comparability. In line with the Outcome Measures in Rheumatology (OMERACT) and EULAR guidance, outcome selection should be domain-informed: skin-focused indices such as PASI are highly responsive for cutaneous disease, whereas ACR20 remains the core joint efficacy endpoint in PsA RCTs (32). Accordingly, when the primary objective is musculoskeletal control, relying on ACR20 (with composite measures such as minimal disease activity (MDA)/Psoriatic Arthritis Disease Activity Score (PASDAS) for broader disease control) helps avoid overestimating overall treatment benefit from skin-dominant responses.

Collectively, these findings highlight the multidimensional approach to efficacy and safety assessment in PsA trials, balancing pharmacokinetic confirmation, biomarker change, composite rheumatologic indices, patient-reported outcomes, and dermatologic measures. This heterogeneity underscores the complexity of PsA and the need for harmonization of endpoint selection across future studies.

Implications. Adopting standardized core outcome sets-such as those promoted by GRAPPA and OMERACT-and consistent use of validated instruments (e.g., MDA, PASDAS) would improve meta-analysis feasibility and comparability. Regulatory bodies and journals should encourage the use of standardized endpoints to accelerate evidence synthesis and guideline development.

### 4.8 Future directions & broader implications

Precision medicine: With diversified targets and trial pipelines, there is opportunity to tailor treatments better—e.g., IL-17 inhibitors for axial/enthesitis-predominant disease, JAK inhibitors for rapid control or bridging regimens.

Safety and regulatory vigilance: Recent JAK inhibitor safety warnings and boxed labels underscore the importance of long-term pharmacovigilance, particularly in PsA populations with comorbidities.

Publication and data transparency: Encouraging full disclosure of trial outcomes—especially “Undefined” or negative results—is critical for balanced evidence and avoiding duplication.

Global inclusivity: Encouraging multicenter, multi-region trials—including beyond traditional Western hubs—will enhance diversity and external validity.

Endpoint consensus: Realignment around core domain-specific outcomes will benefit future comparative effectiveness research and support T2T implementation.

Integration of academia and industry: Sustaining co-funded, multi-sector trials can accelerate innovation while maintaining scientific rigor.

In line with the GRAPPA 2021 update, future research should expand beyond efficacy in core domains to address unmet areas such as tapering strategies, combination regimens with different mechanisms, and special phenotypes including oligoarticular or mutilans PsA. Moreover, improving assessment tools for enthesitis and achieving consensus on the definition and measurement of axial PsA are pressing needs. Trials should also incorporate patient-prioritized outcomes like pain and fatigue, and systematically evaluate long-term safety across therapeutic classes.

## 5 Conclusion

Across 1999–2025, the PsA trial enterprise has shifted from a TNF-centric paradigm to a diversified pipeline spanning IL-17/IL-23, JAK/TYK2, and PDE4 pathways, with sustained phase III/IV activity and higher levels of early-phase exploration in recent years. Geographic patterns remain asymmetric: the U.S. and Europe anchor multicenter collaborations, whereas China has shown a marked increase in single-center programs; these differences may reflect regional epidemiology, regulatory environments, and research infrastructure. Funding is pluralistic: academia (35.3%) and top-20 industry (32.9%) lead, with additional contributions from other pharma (21.6%) and generics (16.4%), suggesting a continued role for co-funded designs that can support head-to-head and pragmatic studies.

Clinically, unmet need persists, particularly for musculoskeletal domains and for patients failing first-line biologic or targeted synthetic DMARDs. Sequencing decisions should weigh domain-specific efficacy, long-term safety (notably with JAK-pathway inhibition), and real-world durability. The growing availability of biosimilars and generics (e.g., adalimumab/ustekinumab biosimilars; tofacitinib and apremilast generics) adds important cost and access considerations that are likely to influence both study design and routine care. Methodologically, heterogeneity in primary endpoints continues to limit cross-trial comparability; broader adoption of harmonized, domain-informed core sets may improve comparability in future syntheses.

Looking ahead, priorities include: (i) domain-stratified, biomarker-guided trials that test sequencing and switching strategies; (ii) long-term safety and comparative effectiveness across mechanisms; (iii) transparent outcome reporting to reduce evidence gaps; and (iv) broader, multi-region networks to improve generalizability. Aligning these efforts with treat-to-target care and evolving policy on biosimilar interchangeability may help speed translation toward effective, affordable, and sustainable PsA therapy.

## 6 Limitations of the study

1. Data source and labeling. Although Trialtrove is a curated and comprehensive resource, outcome tags such as “Undefined” can reflect incomplete public reporting rather than true neutrality, and categorization may differ from systematic-review conventions. Coverage and metadata quality can also vary by region and sponsor. Registry fields (e.g., completion status) may disagree across registries for the same trial.
2. No comparative synthesis. Analyses are descriptive; we did not conduct pairwise or network meta-analysis.
3. Publication and reporting bias. Positive studies are more likely to be published and fully reported.
4. Temporal lag. Pipelines evolve quickly. Although our dataset extends through 2025, subsequent approvals, label changes, and withdrawals may affect the conclusions drawn from this snapshot.
5. Endpoint heterogeneity. The multidomain nature of PsA and evolving outcome frameworks (e.g., MDA, Disease Activity Index for PsA, PASDAS, PsA Impact of Disease 12-item) limit cross-trial comparability across eras and mechanisms, complicating quantitative synthesis and head-to-head inference.
6. Cross-registry triangulation. We did not conduct full independent re-extraction and harmonization across ClinicalTrials.gov, EUCTR/EudraCT/CTIS, and ChiCTR; differences in registry practices and transparency requirements may influence apparent participation and outcomes reporting across countries and eras.
